# Severe Maternal Morbidity and Mortality of Pregnant Patients With COVID-19 Infection During the Early Pandemic Period in the US

**DOI:** 10.1001/jamanetworkopen.2023.7149

**Published:** 2023-04-07

**Authors:** Koji Matsuo, Jessica M. Green, Sarah A. Herrman, Rachel S. Mandelbaum, Joseph G. Ouzounian

**Affiliations:** 1 Division of Gynecologic Oncology, Department of Obstetrics and Gynecology, University of Southern California, Los Angeles; 2 Norris Comprehensive Cancer Center, University of Southern California, Los Angeles; 3 Keck School of Medicine, University of Southern California, Los Angeles; 4 Division of Reproductive Endocrinology & Infertility, Department of Obstetrics and Gynecology, University of Southern California, Los Angeles; 5 Division of Maternal-Fetal Medicine, Department of Obstetrics and Gynecology, University of Southern California, Los Angeles

## Abstract

This cohort study examines the patient characteristics and maternal outcomes associated with COVID-19 infection among pregnant patients at delivery during the early pandemic period in the US.

## Introduction

From the beginning of the COVID-19 pandemic in mid-March, 2020, through mid-February 2023, nearly 103 million cases and 1.1 million deaths were reported in the United States.^1^ Increasing evidence suggests that pregnant patients with COVID-19 infection are at high risk for adverse pregnancy outcomes.^2,3,4^

Several studies have examined outcomes before and during the pandemic periods.^2,3,4^ National-level data comparing pregnancy outcomes with and without COVID-19-infection are limited but are clinically compelling. The objective of this study was to examine the patient characteristics and maternal outcomes associated with COVID-19 infection among pregnant patients at delivery during the early pandemic period in the United States.

## Methods

The University of Southern California institutional review board deemed this cohort study exempt and granted waiver of informed consent because it included only publicly available, deidentified data. This study followed the STROBE reporting guideline.

The Healthcare Cost and Utilization Project’s National Inpatient Sample was retrospectively queried.^5^ The study population was 2 578 095 hospital deliveries across 2691 centers between April and December 2020. The exposure was COVID-19 infection. Race and ethnicity categories in the National Inpatient Sample included American Indian or Alaska Native, Asian, Black, Hispanic, other, and White.

The coprimary end points were (1) patient characteristics associated with COVID-19 infection, assessed with a multivariable binary logistic regression model, and (2) severe maternal morbidity and mortality at delivery associated with COVID-19, assessed with inverse probability of treatment-weighting cohort followed by adjusting for priori obstetric and delivery factors.

Two-tailed *P* < .05 was considered statistically significant. Statistical analysis was performed from November to December 2022 using SPSS Statistics version 28.0 (IBM) and R version 3.5.3 (R Project for Statistical Computing). Additional details about the study methods can be found in eMethods 1, eMethods 2, and eMethods 3 in Supplement 1.

## Results

Among 2 578 095 patients for national estimates included in this study, 5.7% were Asian, 14.7% were Black, 20.6% were Hispanic, and 50.7% were White; the median (IQR) age was 29 (25-33) years (Table 1). During the 9-month study period, 45 425 (17.6 per 1000) pregnant patients had the diagnosis of COVID-19 at delivery and 1704 hospitals (63.3%) cared for pregnant patients with COVID-19 infection at delivery (median, 15 cases). The top-decile centers by volume had 60 or more COVID-19 cases. In multivariable analysis (Table 1), younger age, later study period, Black and Hispanic individuals, lower household income, obesity, medical comorbidity, homelessness status, Northeastern region, earlier gestational age, and hospital admission to larger, urban hospitals were the characteristics associated with COVID-19 infection.

**Table 1.** Patient Characteristics

Characteristic	Patients, No. (%)			aOR (95% CI)^a^	*P* value
	All	COVID-19 negative	COVID-19 positive		
No.	2 578 095	2 532 670	45 425	NA	NA
Age, median (IQR), y	29 (25-33)	29 (25-33)	28 (23-32)	NA	<.001^b^
<20	113 935 (4.4)	110 865 (4.4)	3070 (6.8)	1.32 (1.26-1.38)	<.001
20-24	472 490 (18.3)	461 875 (18.2)	10 615 (23.4)	1.26 (1.22-1.29)	<.001
25-29	724 685 (28.1)	711 385 (28.1)	13 300 (29.3)	1.16 (1.13-1.19)	<.001
30-34	768 000 (29.8)	757 100 (29.9)	10 900 (24.0)	1 [Reference]	NA
35-39	407 740 (15.8)	401 715 (15.9)	6025 (13.3)	1.00 (0.97-1.04)	.84
≥40	91 245 (3.5)	89 730 (3.5)	1515 (3.3)	1.01 (0.95-1.06)	.78
Time					<.001^b^
QT2 (April-June)	849 190 (32.9)	838 455 (33.1)	10 735 (23.6)	1 [Reference]	NA
QT3 (July-September)	898 970 (34.9)	883 250 (34.9)	15 720 (34.6)	1.38 (1.34-1.41)	<.001
QT4 (October-December)	829 935 (32.2)	810 965 (32.0)	18 970 (41.8)	1.79 (1.75-1.84)	<.001
Race and ethnicity					<.001^b^
Asian	148 025 (5.7)	146 425 (5.8)	1600 (3.5)	1.18 (1.12-1.24)	<.001
Black	379 190 (14.7)	371 505 (14.7)	7685 (16.9)	1.51 (1.46-1.56)	<.001
Hispanic	532 350 (20.6)	513 430 (20.3)	18 920 (41.7)	2.97 (2.89-3.04)	<.001
American Indian or Alaska Native	18 835 (0.7)	18 550 (0.7)	285 (0.6)	1.43 (1.27-1.61)	<.001
Other^c^	109 525 (4.2)	106 630 (4.2)	2895 (6.4)	2.34 (2.24-2.44)	<.001
White	1 305 830 (50.7)	1 293 045 (51.1)	12 785 (28.1)	1 [Reference]	NA
Unknown	84 340 (3.3)	83 085 (3.3)	1255 (2.8)	1.36 (1.28-1.44)	<.001
Primary expected payer					<.001^b^
Medicaid	1 069 185 (41.5)	1 042 415 (41.2)	26 770 (58.9)	1 [Reference]	NA
Private including HMO	1 359 975 (52.8)	1 344 435 (53.1)	15 540 (34.2)	0.64 (0.62-0.65)	<.001
Self-pay	57 085 (2.2)	55 375 (2.2)	1710 (3.8)	1.20 (1.14-1.26)	<.001
Medicare	15 740 (0.6)	15 400 (0.6)	340 (0.7)	1.01 (0.91-1.13)	.82
No charge	2040 (0.1)	1955 (0.1)	85 (0.2)	1.20 (0.96-1.49)	.11
Other	71 365 (2.8)	70 445 (2.8)	920 (2.0)	0.68 (0.64-0.73)	<.001
Unknown	2705 (0.1)	2 645 (0.1)	60 (0.1)	1.12 (0.87-1.45)	.38
Household income					<.001 b
QT1 (lowest)	702 120 (27.2)	686 170 (27.1)	15 950 (35.1)	1.31 (1.27-1.35)	<.001
QT2	673 065 (26.1)	660 925 (26.1)	12 140 (26.7)	1.24 (1.20-1.28)	<.001
QT3	615 770 (23.9)	605 250 (23.9)	10 520 (23.2)	1.24 (1.20-1.28)	<.001
QT4 (highest)	569 245 (22.1)	562 730 (22.2)	6515 (14.3)	1 [Reference]	NA
Unknown	17 895 (0.7)	17 595 (0.7)	300 (0.7)	1.09 (0.97-1.23)	.14
Charlson Comorbidity Index					<.001^b^
0	2 340 900 (90.8)	2 300 755 (90.8)	40 145 (88.4)	1 [Reference]	NA
1	200 245 (7.8)	196 010 (7.7)	4235 (9.3)	2.09 (1.99-2.20)	<.001
2	27 370 (1.1)	26 625 (1.1)	745 (1.6)	1.50 (1.39-1.61)	<.001
≥3	9580 (0.4)	9280 (0.4)	300 (0.7)	1.79 (1.59-2.01)	<.001
Hypertensive disorder					<.001^b^
No	2 128 460 (82.6)	2 091 615 (82.6)	36 845 (81.1)	1 [Reference]	NA
Pregestational	64 595 (2.5)	63 605 (2.5)	990 (2.2)	0.83 (0.77-0.88)	<.001
Gestational	174 245 (6.8)	171 600 (6.8)	2645 (5.8)	0.87 (0.83-0.90)	<.001
Preeclampsia	190 140 (7.4)	185 540 (7.3)	4600 (10.1)	1.13 (1.09-1.17)	<.001
NOS	20 655 (0.8)	20 310 (0.8)	345 (0.8)	0.94 (0.85-1.05)	.28
Obesity					
No	2 214 625 (85.9)	2 176 865 (86.0)	37 760 (83.1)	1 [Reference]	NA
Yes	363 470 (14.1)	355 805 (14.0)	7665 (16.9)	1.07 (1.04-1.10)	<.001
Asthma					
No	2 413 250 (93.6)	2 370 565 (93.6)	42 685 (94.0)	1 [Reference]	NA
Yes	164 845 (6.4)	162 105 (6.4)	2740 (6.0)	0.45 (0.43-0.48)	<.001
Tobacco use					
No	2 454 665 (95.2)	2 410 190 (95.2)	44 475 (97.9)	1 [Reference]	NA
Yes	123 430 (4.8)	122 480 (4.8)	950 (2.1)	0.43 (0.40-0.46)	<.001
Prior uterine scar					
No	2 110 040 (81.8)	2 073 195 (81.9)	36 845 (81.1)	1 [Reference]	NA
Yes	468 055 (18.2)	459 475 (18.1)	8580 (18.9)	1.01 (0.98-1.03)	0.57
Homelessness status					
No	2 574 880 (99.9)	2 529 560 (99.9)	45 320 (99.8)	1 [Reference]	NA
Yes	3215 (0.1)	3110 (0.1)	105 (0.2)	1.69 (1.39-2.07)	<.001
Gestational age, wk					<.001^b^
<28	27 155 (1.1)	26 490 (1.0)	665 (1.5)	1.36 (1.26-1.47)	<.001
28-31	23 580 (0.9)	22 885 (0.9)	695 (1.5)	1.58 (1.47-1.71)	<.001
32-33	29 205 (1.1)	28 520 (1.1)	685 (1.5)	1.26 (1.16-1.36)	<.001
34-36	181 775 (7.1)	177 455 (7.0)	4320 (9.5)	1.37 (1.32-1.42)	<.001
37-38	724 765 (28.1)	710 695 (28.1)	14 070 (31.0)	1.19 (1.16-1.21)	<.001
≥39	1 557 470 (60.4)	1 533 160 (60.5)	24 310 (53.5)	1 [Reference]	NA
Unknown	34 145 (1.3)	33 465 (1.3)	680 (1.5)	1.28 (1.19-1.39)	<.001
Hospital bed capacity					<.001^b^
Small	524 884 (20.4)	516 824 (20.4)	8060 (17.7)	1 [Reference]	NA
Medium	747 640 (29.0)	734 485 (29.0)	13 155 (29.0)	1.09 (1.06-1.13)	<.001
Large	1 305 571 (50.6)	1 281 361 (50.6)	24 210 (53.3)	1.21 (1.18-1.24)	<.001
Hospital location/teaching					<.001^b^
Rural	225 031 (8.7)	222 541 (8.8)	2490 (5.5)	1 [Reference]	NA
Urban nonteaching	439 601 (17.1)	433 376 (17.1)	6225 (13.7)	1.21 (1.15-1.27)	<.001
Urban teaching	1 913 463 (74.2)	1 876 753 (74.1)	36 710 (80.8)	1.51 (1.45-1.58)	<.001
Hospital region					<.001^b^
Northeast	412 245 (16.0)	403 660 (15.9)	8585 (18.9)	1 [Reference]	NA
Midwest	538 966 (20.9)	530 486 (20.9)	8480 (18.7)	0.90 (0.87-0.93)	<.001
South	1 027 035 (39.8)	1 008 095 (39.8)	18 940 (41.7)	0.79 (0.77-0.82)	<.001
West	599 849 (23.3)	590 429 (23.3)	9420 (20.7)	0.61 (0.59-0.63)	<.001

Abbreviations: aOR, adjusted odds ratio; HMO, Health Maintenance Organization; NA, not applicable; NOS, not otherwise specified; QT, quarter.

^a^ A multivariable binary logistic regression model. All the listed priori selected maternal factors in the table were entered in the modeling. Results were similar when unknown cases were excluded in sensitivity analysis.

^b^ Overall *P* value.

^c^ Designated per the National Inpatient Sample.

Pregnant patients with COVID-19 infection at delivery were more likely to develop severe maternal morbidity compared with those without (46.4 vs 18.8 per 1000, adjusted odds ratio [aOR]: 2.60 [95% CI, 2.39-2.82]) (Table 2). Among the individual morbidity indicators, COVID-19 infection was associated with the following outcomes with all aORs greater than 2: increased risk of tracheostomy, respiratory distress syndrome, ventilation, acute myocardial infarction, sepsis, shock, cardiac arrest, and coagulopathy (Table 2).

**Table 2.** Severe Maternal Morbidity and Mortality

Parameter	Outcome rate (per 1000)^a^		IPTW cohort^b^	
	COVID-19 negative	COVID-19 positive	aOR (95% CI)^c^	*P* value
Severe maternal morbidity (composite)				
Any^d^	18.8	46.4	2.60 (2.39-2.82)	<.001
Except for blood transfusion	7.6	33.4	4.69 (4.25-5.18)	<.001
Except for blood transfusion/hysterectomy	7.1	32.6	4.77 (4.31-5.27)	<.001
Core morbidity^e^	2.5	27.0	11.42 (10.15-12.84)	<.001
Individual indicator^f^				
Temporary tracheostomy	<0.1	0.6	35.76 (13.68-93.49)	<.001
Adult respiratory distress syndrome	0.9	24.0	28.71 (24.85-33.18)	<.001
Ventilation	0.5	7.8	17.65 (14.02-22.22)	<.001
Acute myocardial infarction	<0.1	0.6	8.60 (4.00-18.52)	<.001
Sepsis	1.2	7.5	6.53 (5.30-8.04)	<.001
Shock	0.8	2.9	3.70 (2.67-5.12)	<.001
Cardiac arrest/ventricular fibrillation	<0.1	0.4	3.65 (1.40-9.50)	.008
Disseminated intravascular coagulation	1.8	4.9	2.68 (2.09-3.43)	<.001
Hysterectomy	0.7	1.1	1.87 (1.04-3.35)	.04
Pulmonary edema	0.9	1.6	1.83 (1.20-2.81)	.005
Acute kidney failure	1.9	3.1	1.62 (1.19-2.20)	.002
Eclampsia	0.8	1.4	1.56 (1.00-2.53)	.05
Blood products transfusion	12.6	18.5	1.48 (1.30-1.68)	<.001
Air/thrombotic embolism	0.3	0.4	1.24 (0.53-2.89)	.63
Other outcome measures				
Death (per 100 000)^g^	4.3	64.0	13.91 (6.36-30.42)	<.001
Failure-to-rescue, %^h^	0.2	1.5	5.56 (2.51-12.30)	<.001
Hospital admission ≥7 d	14.4	28.1	1.98 (1.79-2.20)	<.001
Race and ethnicity^d^^,^^i^				
Asian	20.8	48.0	4.37 (3.26-5.86)	<.001
Black	29.4	62.6	2.25 (1.88-2.68)	<.001
Hispanic	20.8	48.0	2.46 (2.06-2.93)	<.001
White	14.7	36.6	2.60 (2.28-2.97)	<.001

Abbreviations: aOR, adjusted odds ratio; IPTW, inverse probability of treatment weighting.

^a^ Outcome rates per 1000 deliveries in the IPTW cohort except for mortality and failure-to-rescue rates.

^b^ IPTW cohort: Covariates listed in Table 1 were considered. Stabilization method was applied, and a trimming technique at 10 was used. Measured covariates were well balanced in the IPTW cohort.

^c^ IPTW-adjusted model: Adjusted for priori selected obstetric and delivery factors known for severe maternal morbidity that were not used in the IPTW modeling: cesarean delivery, placenta abruption, placenta accreta spectrum, and uterine rupture. Effect size of the exposure group (COVID-19 compared with non–COVID-19) on outcome measures (maternal morbidity and mortality) is shown as aOR, and the non–COVID-19 group served as the referent. The increased risk of severe maternal morbidity in COVID-19 was robust in several sensitivity analyses: exclusion of unknown cases, standard logistic regression model, cesarean delivery, and vaginal delivery (data not shown).

^d^ Included any one of the 21 indicators for severe maternal morbidity per the Centers for Disease Control and Prevention definition.

^e^ Any of 8 morbidity indicators exhibiting aOR of >2. Advanced maternal age, early time, Asian and American Indian or Alaska Native individuals, higher comorbidity, pregestational hypertension, obesity, homelessness status, early gestational age were associated with increased risk of core morbidity among pregnant patients with COVID-19 infection.

^f^ Individual morbidity indicator is listed in the descending order of effect size. Indicators with no or small event number in the COVID-19 group are not listed.

^g^ Inpatient mortality event rate is shown per 100 000 deliveries.

^h^ Mortality rate among severe maternal morbidity.

^i^ Four most frequent race and ethnicity groups were examined.

The mortality risk of pregnant patients with COVID-19 infection at delivery was approximately 14 times higher compared with those without (64.0 vs 4.3 per 100 000 deliveries; aOR, 13.91 [95% CI, 6.36-30.42] (Table 2). Failure-to-rescue risk following the development of severe maternal morbidity was also increased in pregnant patients with COVID-19 infection at delivery (1.5% vs 0.2%; aOR, 5.56 [95% CI, 2.51-12.30]).

Among the pregnant patients with COVID-19 infection who died during the hospital admission, the median time-to-death was 16 days, but this was particularly short in the early period (median 6 days in April to June 2020). The COVID-19 case-fatality rate decreased over time from 232.9 to 79.1 per 100 000 deliveries (*P* for trend < .001).

## Discussion

This national-level analysis found substantial adverse maternal outcomes among pregnant patients with COVID-19 infection at delivery during the early pandemic in the US. Specifically, the odds of severe respiratory complications were increased among pregnant patients with COVID-19 infection at delivery. Greater than 10-fold increased maternal mortality risk associated with COVID-19 infection validates prior investigations with national-level data^6^; moreover, increased failure-to-rescue risk following severe maternal morbidity among patients with COVID-19 adds important information.

Key limitations included lack of information on COVID-19 infection status (eg, severity and treatment), neonatal outcomes, delivery indication, and cause of death. Despite these limitations, the evaluation of the initial pandemic period in this study demonstrates the substantial morbidity and mortality of COVID-19 in pregnant patients and highlights the importance of prevention of COVID-19 in this population. Further investigation to assess the generalizability in the subsequent vaccine era is warranted.

## Notes

Supplement 1. **eMethods 1.** Description of Analysis **eMethods 2.** Code Information **eMethods 3.** Balance Statistics

Supplement 2.
Data Sharing Statement
