# Supplementary material for: Severe Maternal Morbidity and Mortality of Pregnant Patients With COVID-19 Infection During the Early Pandemic Period in the US
Source: JAMA Netw Open. 2023 Apr 7;6(4):e237149. doi: 10.1001/jamanetworkopen.2023.7149 (PMC10082398; doi:10.1001/jamanetworkopen.2023.7149)
Supplement: Supplement 1. — eMethods 1. Description of Analysis eMethods 2. Code Information eMethods 3. Balance Statistics [file jamanetwopen-e237149-s001.pdf]

Supplemental Online Content

Matsuo K, Green JM, Herrman SA, Mandelbaum RS, Ouzounian JG. Severe maternal morbidity and mortality of pregnant patients with COVID-19 infection during the early pandemic period in the US. *JAMA Netw Open.* 2023;6(4):e237149. doi:10.1001/jamanetworkopen.2023.7149

eMethods 1. Description of Analysis

eMethods 2. Code Information

eMethods 3. Balance Statistics

This supplemental material has been provided by the authors to give readers additional information about their work.

© 2023 Matsuo K et al. *JAMA Network Open.*

eMethods 1. Description of analysis.

1.1. Data

The Healthcare Cost and Utilization Project's National Inpatient Sample was retrospectively queried.[1] Their project is supported and distributed by the Agency for Healthcare Research and Quality. The program approximates a stratified-sample of 20% of discharges in each center from all the participating hospitals across 48 States and the District of Columbia every year. By applying the survey weights, the National Inpatient Sample represents more than 90% of hospital discharge data in the U.S. population. The University of Southern California Institutional Review Board exempted the current study due to the use of publicly available, deidentified data.

1.2. Study population

The study population included hospital-setting vaginal and cesarean deliveries across 2,691 centers between April 1, 2020 and December 31, 2020. Identification of vaginal and cesarean deliveries followed prior studies based on the Diagnosis-Related Group, World Health Organization's International Classification of Disease 10th revision Clinical Modification (ICD-10-CM), and ICD-10 Procedure Coding System (ICD-10-PCS) codes (eMethod 2).[2-4] Patient age were limited to 15-54 years according to prior analysis.[5,6]

1.3. Exposure

The exposure was based on the ICD-10-CM code of U07.1 for COVID-19 diagnosis according to the Centers for Disease Control and Prevention (CDC)'s recommendation.[7] Pregnant patients with this code were assigned as the COVID-19 group, and those without were assigned as the non-COVID-19 group. The ICD-10 code was consistent throughout the study period.

The National Inpatient Sample captures maximum 40 diagnoses via ICD-10-CM schema for each patient during the index hospital admission. The National Inpatient Sample captures the information for the index hospitalization only, but not post-discharge information. While the identification of COVID-19 was only relied on the ICD-10-CM coding without exact medical record review to ensure the diagnosis of COVID-19 infection during the hospitalization, the accuracy of this code has been externally validated previously.[8]

1.4. Outcome measures

The co-primary endpoints were *(i)* patient characteristics associated with COVID-19 infection and *(ii)* severe maternal morbidity and mortality at delivery associated with COVID-19.

Identification of sever maternal morbidity followed the CDC criteria (a total of 21 indicators).[9] The These included (A-Z): acute myocardial infarction, acute renal failure, adult respiratory distress syndrome, air and thrombotic embolism, amniotic fluid embolism, aneurysm, blood products transfusion, cardiac arrest / ventricular fibrillation, cardiac rhythm conversion, disseminated intravascular coagulation, eclampsia, heart failure / arrest during surgery or procedure, hysterectomy, puerperal cerebrovascular disorders, pulmonary edema / acute heart failure, severe anesthesia complications, sepsis, shock, sickle cell disease with crisis, temporary tracheostomy, and ventilation. The ICD-10-CM and ICD-10-PCS codes were used to identify these morbidity indicators (eMethod 2).

The National Inpatient Sample captures maximum 40 diagnoses and 25 procedures during the index admission for each patient. The National Inpatient Sample captures the mortality event occurred during the index hospital admission. These data following discharge from the index hospital admission are not available in the National Inpatient Sample.

1.5. Study covariates

The study covariates examined were pre-selected in a view of relevance to the study subjects. These included patient age (<20, 20-24, 25-29, 30-34, 35-39, and ≥40 years), time period per year-quarter (April-June, 2020; July-September, 2020; and October-December, 2020), race and ethnicity (A-Z: Asian, Black, Hispanic, Native American, other, and White) determined by the National Inpatient Sample, primary

© 2023 Matsuo K et al. *JAMA Network Open.*

expected payer (Medicaid, private including HMO, self-pay, Medicare, no charge, and other), census-level median household income (every quartile), Charlson Comorbidity Index (0, 1, 2, and ≥3) calculated for each patient based on the codes for the specified medical conditions in each category and weighted appropriately to calculate a final score (eMethod 2),[10] hypertensive disorder (pregestational, gestational, or pre-eclampsia), obesity (yes or no), asthma (yes or no), tobacco use (yes or no), homelessness status (yes or no),[11] and gestational age (<28, 28-31, 32-33, 34-36, 37-38, and ≥39 weeks) grouped per prior analysis.[12] Race and ethnicity was examined as this information is associated with pregnancy and COVID-19 characteristics and outcomes.

Hospital delivery factors included hospital relative bed capacity (small, mid, and large), hospital location and teaching status (rural, urban non-teaching, and urban teaching), and hospital region (Northeast, Midwest, South and West). These hospital parameters were determined and grouped by the National Inpatient Sample.

The identification of these study covariates other than information provided by the National Inpatient Sample was based on the ICD-10-CM codes (eMethod 2). The study covariates were consistent during the study period and grouped as similar to prior studies.[2-4,12]

1.6. Analytic consideration

The first step of analysis was to estimate the incidence rate of pregnant patients with diagnosis of COVID-19 at hospital-setting delivery. The rates were generated per 1,000 hospital deliveries. Information on hospital treatment volume for pregnant patients with COVID-19 diagnosis at delivery was also described.

The second step of analysis was to identify the independent clinical characteristics associated with COVID-19 infection. A binary logistic regression model was fitted for multivariable analysis. All the measured covariates were modeled in the analysis. Multicollinearity was assessed among the study covariates. The effect size for COVID-19 infection was estimated with adjusted-odds ratio (aOR) and a corresponding 95% confidence interval (CI).

The third step of analysis was to assess the delivery outcomes of pregnant patients with a diagnosis of COVID-19 infection at delivery. The baseline confounders between the two exposure groups (COVID-19 and non-COVID-19) were balanced by creating the inverse probability of treatment weighting (IPTW) cohort.[13] Propensity score was computed by modeling the measured study covariates. The IPTW propensity score method assigned patients in the COVID-19 group a weight of 1 / (propensity score) and those in the non-COVID-19 group a weight of 1 / (1-propensity score).

Stabilized weights and threshold technique at 10 were used. In the IPTW cohort, balance statistics between the two exposure groups were assessed with standardized difference, and a value greater than 0.20 was interpreted as clinical imbalance between the exposure groups.[14] The measured study covariates in the IPTW cohort were all well-balanced clinically (eMethod 3).

The exposure-outcome association was assessed in the IPTW cohort to account for the baseline confounders, followed by adjusting for the *priori* selected obstetric and delivery factors known for severe maternal morbidity that were not used in the IPTW modeling. These included cesarean delivery, placenta abruption, placenta accreta spectrum, and uterine rupture. Effect size of the exposure group (COVID-19 compared to non-COVID-19) on outcome measures (severe maternal morbidity and mortality) was estimated as aOR and a corresponding 95%CI.

Various sensitivity analyses were undertaken to assess the robustness of study findings. First, maternal outcomes were assessed as: panel of 21 indicators, except for blood transfusion, except for blood transfusion and hysterectomy as similar to the CDC analysis.[15] Outcomes were also assessed in each morbidity indicator. Core morbidity indicator was defined as the severe maternal morbidity that had effect size of greater than twofold.

© 2023 Matsuo K et al. *JAMA Network Open.*

Second, study cohort was restricted by excluding 5.3% of cases that had unknown data in study covariates. Third, standard logistic regression was fitted to assess the exposure-outcome association, adjusting for study covariates and priori selected obstetric and delivery factors. Fourth, the exposure-outcome association was assessed in cesarean delivery cases or vaginal delivery cases separately. Fifth, race and ethnicity-specific analysis was performed given disparity per this factor.[16,17] Sixth, detailed of mortality data were assessed including time to death and cohort-level mortality trends. Last, failure-to-rescue, defined as mortality following severe maternal morbidity, was assessed.[3,17]

In an exploratory analysis, patient clinical characteristics were assessed among the pregnant patients with a diagnosis of COVID-19 infection at delivery who developed the core morbidity (aOR >2). Patients with placenta abruption, placenta accreta spectrum, and uterine rupture were excluded in this exploratory analysis.

The weights for national estimates provided by the National Inpatient Sample were used for analysis. Statistical interpretation followed a two-tailed hypothesis, and a *P*-value of less than 0.05 was considered statistically significant. Cases with unknown data were grouped as one category in each variable. IBM SPSS Statistics (version 28.0, Armonk, NY, USA) and R version 3.5.3 (R Foundation for Statistical Computing, Vienna, Austria) were used for all analysis. This study followed the STROBE reporting guidelines to summarize the performance of the cohort study.[18]

1.7. References

1. Overview of the National (Nationwide) Inpatient Sample (NIS). Agency for Healthcare Research and Quality. https://www.hcup-us.ahrq.gov/nisoverview.jsp.
2. Matsuzaki S, Mandelbaum RS, Sangara RN, et al. Trends, characteristics, and outcomes of placenta accreta spectrum: a national study in the United States. *Am J Obstet Gynecol.* 2021;225(5):534 e531-534 e538.
3. Matsuo K, Klar M, Youssefzadeh AC, et al. Assessment of Severe Maternal Morbidity and Mortality in Pregnancies Complicated by Cancer in the US. *JAMA Oncol.* 2022;8(8):1213-1216.
4. Mazza GR, Youssefzadeh AC, Klar M, et al. Association of Pregnancy Characteristics and Maternal Mortality With Amniotic Fluid Embolism. *JAMA Netw Open.* 2022;5(11):e2242842.
5. Logue TC, Wen T, Monk C, et al. Trends in and complications associated with mental health condition diagnoses during delivery hospitalizations. *Am J Obstet Gynecol.* 2022;226(3):405 e401-405 e416.
6. Youssefzadeh AC, Tavakoli A, Panchal VR, Mandelbaum RS, Ouzounian JG, Matsuo K. Incidence trends of shoulder dystocia and associated risk factors: A nationwide analysis in the United States. *Int J Gynaecol Obstet.* 2023.
7. Centers for Disease Control and Prevention. New ICD-10-CM code for the 2019 Novel Coronavirus (COVID-19), April 1, 2020. Available at: https://www.cdc.gov/nchs/data/icd/Announcement-New-ICD-code-for-coronavirus-3-18-2020.pdf. .
8. Bhatt AS, McElrath EE, Claggett BL, et al. Accuracy of ICD-10 Diagnostic Codes to Identify COVID-19 Among Hospitalized Patients. *J Gen Intern Med.* 2021;36(8):2532-2535.
9. How does CDC identify severe maternal morbidity? Centers for Disease Control and Prevention. <<https://www.cdc.gov/reproductivehealth/maternalinfanthealth/smm/severe-morbidity-ICD.htm>>.
10. Charlson ME, Pompei P, Ales KL, MacKenzie CR. A new method of classifying prognostic comorbidity in longitudinal studies: development and validation. *J Chronic Dis.* 1987;40(5):373-383.
11. Porter NAC, Brosnan HK, Chang AH, Henwood BF, Kuhn R. Race and Ethnicity and Sex Variation in COVID-19 Mortality Risks Among Adults Experiencing Homelessness in Los Angeles County, California. *JAMA Netw Open.* 2022;5(12):e2245263.
12. Youssefzadeh AC, Mazza GR, Mandelbaum RS, Ouzounian JG, Matsuo K. Trends of preterm delivery in the United States, 2016-2019. AJOG Glob Rep 2023, in-press.

13. Austin PC, Stuart EA. Moving towards best practice when using inverse probability of treatment weighting (IPTW) using the propensity score to estimate causal treatment effects in observational studies. *Stat Med.* 2015;34(28):3661-3679.
14. Cohen J. *Statistical power analysis for the behavioral sciences*. 2nd ed. L. Erlbaum Associates; 1988:xxi, 567 p.
15. Severe Maternal Morbidity in the United States. Centers for Disease Control and Prevention. https://www.cdc.gov/reproductivehealth/maternalinfanthealth/severematernalmorbidity.html.
16. Guglielminotti J, Wong CA, Friedman AM, Li G. Racial and Ethnic Disparities in Death Associated With Severe Maternal Morbidity in the United States: Failure to Rescue. *Obstet Gynecol.* 2021;137(5):791-800.
17. Matsuo K, Mandelbaum RS, Matsuzaki S, et al. Decreasing Failure-to-Rescue From Severe Maternal Morbidity at Cesarean Delivery: Recent US Trends. *JAMA Surg.* 2021;156(6):585-587.
18. Ghaferi AA, Schwartz TA, Pawlik TM. STROBE Reporting Guidelines for Observational Studies. *JAMA Surg.* 2021;156(6):577-578.

© 2023 Matsuo K et al. *JAMA Network Open.*

eMethods 2. Code information.

	DRG	ICD-10 CM	ICD-10 PCS
COVID-19		U071	
Cesarean section	765, 766, 783, 784, 785, 786, 787, 788	O82, O7582	10D00Z0, 10D00Z1, 10D00Z2
Vaginal delivery	767, 768, 774, 775, 796, 797, 798, 805, 806, 807	O80	10D07Z3, 10D07Z4, 10D07Z5, 10D07Z6, 10D07Z7, 10D07Z8
**Charlson Comorbidity Index**			
CC1: Myocardial Infarction		I21, I22, I23, I252, I9771	
CC2: Congestive Heart Failure		I50, I42, I110, I130, I132, I0981, I9713	
CC3: Peripheral Vascular Disease		I70, I71, I72, I73, I74, I75, I76, I77, I78, I80, I81, I82, I83, I86, I87, K64	
CC4: Cerebrovascular Disease		G45, G46, H34, I60, I61, I62, I63, I64, K65, I66, I67, I68, I69	
CC5: Dementia		F01, F02, F03, F04, G30, G31, F1027, F1097, F1327, F1397, F1817, F1897, F1917, F1997,	
CC6: Chronic Pulmonary Disease		I27, J17, J40, J41, J42, J43, J44, J45, J46, J47, J60, J61, J62, J63, J64, J65, J66, J67, J68, J69, J70, J84, J85, J86, J93, J98, J811, R091	
CC7: Connective Tissue Disease		M04, M05, M06, M08, M10, M11, M12, M13, M30, M31, M32, M33, M34, M35, M36, L94	
CC8: Peptic Ulcer Disease		K25, K26, K27, K28, K221, K633	
CC9: Mild Liver Disease		B18, K73, K74, K700, K758, K760, K752, K753, K758, K759, K760, K761, K769, K77	
CC10: Diabetes without complications		E089, E099, E109, E119, E139	
CC11: Diabetes with complications		E080, E081, E082, E083, E084, E085, E086, E088, E090, E091, E092, E093, E094, E095, E096, E098, E100, E101, E102, E103, E104, E105, E106, E108, E110, E111, E112, E113, E114, E115, E116, E118, E130, E131, E132, E133, E134, E135, E136, E138	
CC12: Paraplegia and hemiplegia		G80, G81, G82, G041, G114, G434, G801, G802, G822, G830, G831, G832, G833, G834, G838, G839, M623, I6925, I6935, I6985, I6995, I6996	
CC13: Renal disease		I12, I13, N00, N01, N02, N03, N04, N05, N07, N11, N13, N15, N16, N17, N18, N19, N20, N25, N26, N27, N28, N28, Q61, Z49	
CC14: Cancer		C01-C99	
CC15: Moderate or Severe Liver Disease		K701, K702, K703, K704, K709, K71, K72, K750, K751, K754, K762,	

© 2023 Matsuo K et al. *JAMA Network Open.*

		K763, K764, K765, K766, K767, K768	
CC16: Metastatic Carcinoma		C77, C78, C79, C80, C7B	
CC17: HIV/AIDS		B20, R75, Z21, B9735	
Obesity		E660, E661, E668, E669, Z683, O9921<br>E662, Z684, E6601	
Pregnancy Hypertensive Disorders		O10, O11, O13, O14, O15, O16	
Tobacco Use		Z720, O9933, F17, T652	
Asthma		J45	
Prior uterine scar		O342	
Homeless		Z590	
Gestational Age		Z3A	
Placenta accreta spectrum		O432	
Placenta Previa		O440, O441, O442, O443	
Placental Abruption		O45	
Uterine rupture		O710, O711	
**CDC Severe Maternal Morbidity**			
Acute myocardial infarction		I21, I22	
Aneurysm		I71, I79	
Acute renal failure		N170, N171, N172, N178, N179, O904	
Adult respiratory distress syndrome		J80, J951, J952, J95821, J95822, J953, J9600, J9601, J9602, J9620, J9621, J9622, R092	
Amniotic fluid embolism		O881	
Cardiac arrest/ventricular fibrillation		I462, I468, I469, I4901, I4902	
Conversion of cardiac rhythm			5A2204Z, 5A12012
Disseminated intravascular coagulation		D65, D688, D689, O723	
Eclampsia		O15	
Heart failure/arrest during surgery or procedure		I97120, I97121, I97130, I97131, I97710, I97711	
Puerperal cerebrovascular disorders		I600, I601, I602, I603, I604, I605, I606, I607, I608, I609, I611, I612, I613, I614, I615, I616, I617, I618, I619, I620, I621, I629, I630, I631, I632, I633, I634, I635, I636, I638, I639, I650, I651, I652, I658, I659, I660, I661, I662, I668, I669, I670, I671, I672, I673, I674, I675, I676, I677, I678, I679, I680, I682, I688, O873, O2251, O2252, O2253, I97800, I97811, I97820, I97821,	
Pulmonary edema / Acute heart failure		J810, I501, I5020, I5021, I5023, I5030, I5031, I5033, I5040, I5041, I5043, I509	
Severe anesthesia complications		O740, O741, O742, O743, O8901, O8909, O891, O892	
Sepsis		O85, O8604, A327, A400, A401, A403, A408, A409, A419, A4101, A4102, A411, A412, A413, A414, A4150, A4151, A4152, A4153, A4159, A4181, A4189, R6520,	

		T8144, T80211A, T814XXA, T8144XA, T8144XD, T8144XS	
Shock		O751, R570, R571, R578, R579, R6521, T782XXA, T882XXA, T886XXA, T8110XA, T8111XA, T8119XA	
Sickle cell disease with crisis		D5700, D5701, D5702, D57211, D57212, D57219, D57411, D57412, D57419, D57811, D57812, D57819	
Air and thrombotic embolism		I2601, I2602, I2609, I2690, I2692, I2699, 08802, O8803, O8822, O8823, O8832, O8833, O8881, O8882, O8883, O88111, O88112, O88113, O88114, O88115, O88116, O88117, O88118, O88119, O88211, O88212, O88213, O88214, O88215, O88216, O88217, O88218, O88219, O88311, O88312, O88313, O88314, O88315, O88316, O88317, O88318, O88319	
Blood products transfusion			30233H1, 30233L1, 30233K1, 30233M1, 30233N1, 30233P1, 30233R1, 30233T1, 30233H0, 30233L0, 30233K0, 30233M0, 30233N0, 30233P0, 30233R0, 30233T0, 30230H1, 30230L1, 30230K1, 30230M1, 30230N1, 30230P1, 30230R1, 30230T1, 30230H0, 30230L0, 30230K0, 30230M0, 30230N0, 30230P0, 30230R0, 30230T0, 30240H1, 30240L1, 30240K1, 30240M1, 30240N1, 30240P1, 30240R1, 30240T1, 30240H0, 30240L0, 30240K0, 30240M0, 30240N0, 30240P0, 30240R0, 30240T0, 30243H1, 30243L1, 30243K1, 30243M1, 30243N1, 30243P1, 30243R1, 30243T1, 30243H0, 30243L0, 30243K0, 30243M0, 30243N0, 30243P0, 30243R0, 30243T0, 30250H1, 30250L1, 30250K1, 30250M1, 30250N1, 30250P1, 30250R1, 30250T1, 30250H0, 30250L0, 30250K0, 30250M0, 30250N0, 30250P0,

© 2023 Matsuo K et al. *JAMA Network Open.*

			30250R0, 30250T0, 30253H1, 30253L1, 30253K1, 30253M1, 30253N1, 30253P1, 30253R1, 30253T1, 30253H0, 30253L0, 30253K0, 30253M0, 30253N0, 30253P0, 30253R0, 30253T0, 30260H1, 30260L1, 30260K1, 30260M1, 30260N1, 30260P1, 30260R1, 30260T1, 30260H0, 30260L0, 30260K0, 30260M0, 30260N0, 30260P0, 30260R0, 30260T0, 30263H1, 30263L1, 30263K1, 30263M1, 30263N1, 30263P1, 30263R1, 30263T1, 30263H0, 30263L0, 30263K0, 30263M0, 30263N0, 30263P0, 30263R0, 30263T0
Hysterectomy			0UT90ZZ, 0UT94ZZ, 0UT97ZZ, 0UT98ZZ, 0UT9FZZ, 0UB9, 0UT9
Temporary tracheostomy			0B110Z4, 0B110F4, 0B113Z4, 0B113F4, 0B114Z4, 0B114F4
Ventilation			5A1935Z, 5A1945Z, 5A1955Z

© 2023 Matsuo K et al. *JAMA Network Open.*

eMethods 3. Balance statistics.

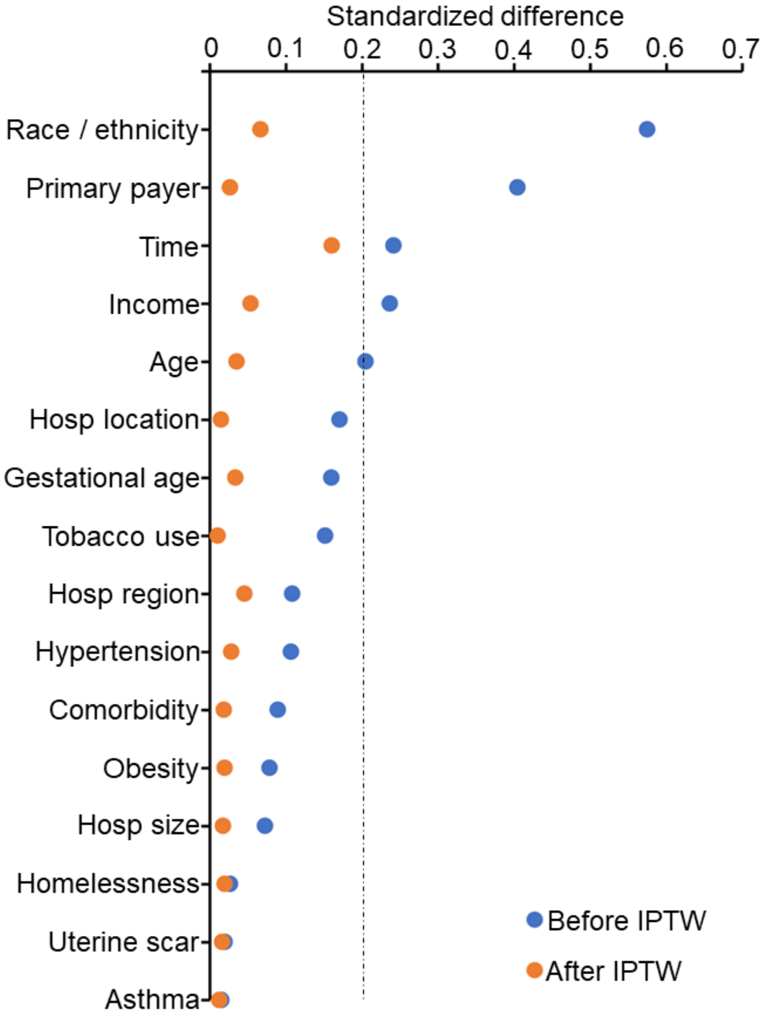

© 2023 Matsuo K et al. *JAMA Network Open.*
