# Supplementary material for: Severe Maternal Morbidity and Mortality of Pregnant Patients With COVID-19 Infection During the Early Pandemic Period in the US
Source: JAMA Netw Open. 2023 Apr 7;6(4):e237149. doi: 10.1001/jamanetworkopen.2023.7149 (PMC10082398; doi:10.1001/jamanetworkopen.2023.7149)
Supplement: Supplement 2. — Data Sharing Statement [file jamanetwopen-e237149-s002.pdf]

Data Sharing Statement

Matsuo. Severe Maternal Morbidity and Mortality of Pregnant Patients With COVID-19 Infection During the Early Pandemic Period in the US. *JAMA Netw Open*. Published April 07, 2023. doi:10.1001/jamanetworkopen.2023.7149

Data
Data available: No

Additional Information
Explanation for why data not available: Data availability statement: The data on which this study is based are publicly available upon request at Healthcare Cost and Utilization Project, Agency for Healthcare Research and Quality. https://www.hcup-us.ahrq.gov/nisoverview.jsp
